# Supplementary material for: Clinical Outcome of Nivolumab Plus Ipilimumab in Patients with Locally Advanced Non-Small-Cell Lung Cancer with Relapse after Concurrent Chemoradiotherapy followed by Durvalumab
Source: Cancers (Basel). 2024 Apr 3;16(7):1409. doi: 10.3390/cancers16071409 (PMC11011053; doi:10.3390/cancers16071409)
Supplement: Supplementary file 1 [file cancers-16-01409-s001.zip › Supplementary Figures S1 and S2.pdf]

Supplementary Figure S1. Swimmer plots in patients who received nivolumab plus ipilimumab to relapsing after concurrent chemoradiotherapy followed by consolidation durvalumab.

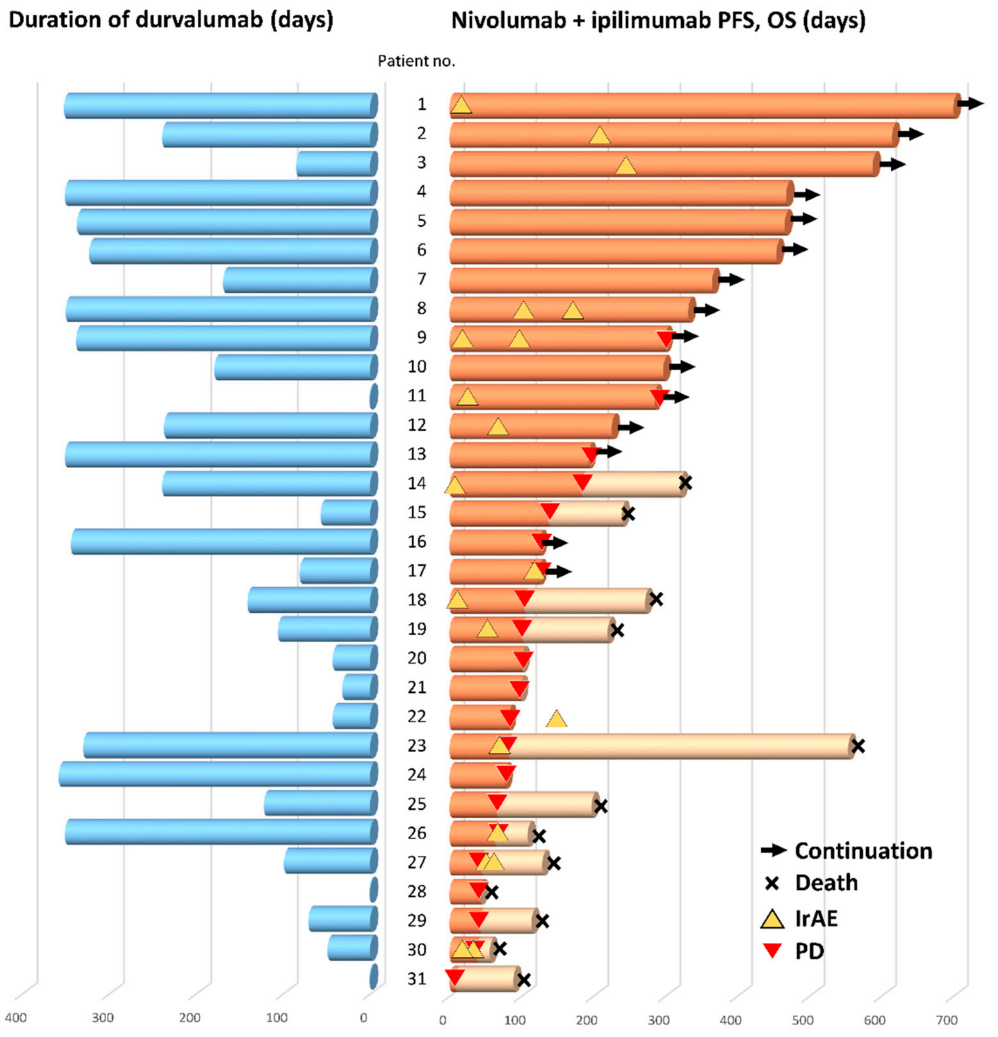

Figure S1: Swimmer plots in patients who received nivolumab plus ipilimumab to relapsing after concurrent chemoradiotherapy followed by consolidation durvalumab. The blue bar on the left shows the duration of treatment with durvalumab, the orange bar on the right shows the progression free survival for nivolumab and ipilimumab, and the light orange shows overall survival after completion of treatment.
PFS: progression free survival, OS: overall survival, PD: progressive disease.

Supplementary Figure S2. Kaplan–Meier curves for time from completion of CRT to recurrence in 3 groups: within 6 months, 6–12 months, and more than 12 months.

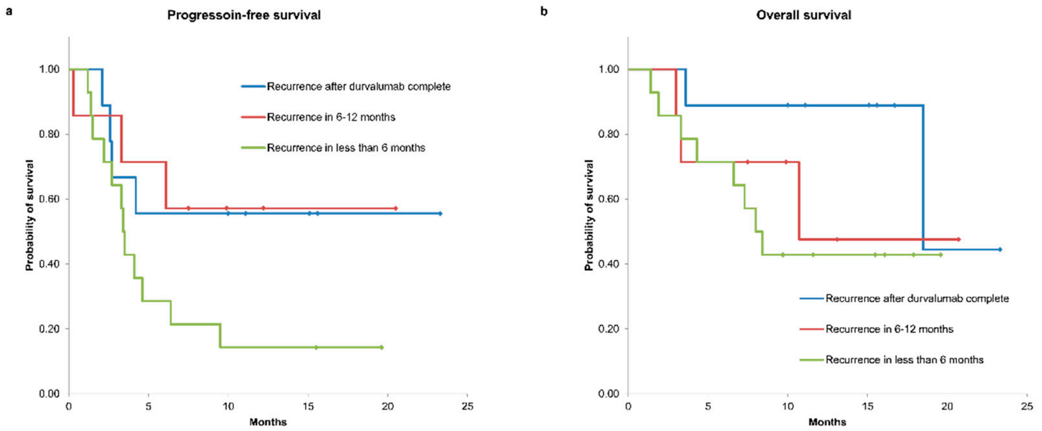

Figure S2: Kaplan-Meier curves for time from completion of CRT to recurrence in 3 groups: within 6 months, 6-12 months, and more than 12 months The blue line was recurrence after durvalumab completion one year (n = 9), the red line was recurrence in 6-12 months after completion of radiotherapy (n = 7), and the green line was recurrence within 6 months after completion of radiotherapy (n = 14)
