# Supplementary material for: Clinical Outcome of Nivolumab Plus Ipilimumab in Patients with Locally Advanced Non-Small-Cell Lung Cancer with Relapse after Concurrent Chemoradiotherapy followed by Durvalumab
Source: Cancers (Basel). 2024 Apr 3;16(7):1409. doi: 10.3390/cancers16071409 (PMC11011053; doi:10.3390/cancers16071409)
Supplement: Supplementary file 1 [file cancers-16-01409-s001.zip › Supplementary Table S1.pdf]

Supplementary Table S1. Therapeutic profiles of CCRT followed by durvalumab consolidation

**Chemoradiotherapy phase**		n, (%)
**Radiotherapy**	Median total radiation, (range)	60 Gy (45-60)
	Median total fraction, (range)	30 Fr (15-30)
**Chemotherapy regimen**	Weekly CBDCA+PTX	12 (40)
	Low dose CBDCA	7 (23.3)
	CDDP+DTX	3 (10)
	CBDCA+PTX	3 (10)
	CDDP+S-1	3 (10)
	CDDP+PEM	1 (3.3)
	CDDP+ETP	1 (3.3)
**Durvalumab consolidation phase**		
**Completion or withdrawal of durvalumab administration**	Completion of durvalumab	10 (33.3)
	Discontinuation due to AEs	3 (10)
	Discontinuation due to PD	16 (19.7)
	Others	1 (3.3)

CCRT, concurrent chemoradiotherapy; Gy, gray; Fr, fractions; CBDCA, carboplatin; PTX, paclitaxel; CDDP, cisplatin; DTX, docetaxel; PEM, pemetrexed; ETP, etoposide; AE, adverse event; PD, progressive disease.
