# Supplementary material for: Clinical Outcome of Nivolumab Plus Ipilimumab in Patients with Locally Advanced Non-Small-Cell Lung Cancer with Relapse after Concurrent Chemoradiotherapy followed by Durvalumab
Source: Cancers (Basel). 2024 Apr 3;16(7):1409. doi: 10.3390/cancers16071409 (PMC11011053; doi:10.3390/cancers16071409)
Supplement: Supplementary file 1 [file cancers-16-01409-s001.zip › Supplementary Table S2.pdf]

Supplementary Table S2. Adverse events with durvalumab consolidation

Variable	All grade n, (%)	≥ grade 3 n, (%)
Hyper or hypothyroidism	4 (13.3)	0
Pneumonitis	18 (60)	3 (10)
Liver dysfunction	1 (3.3)	0
Skin disorder	4 (13.3)	0
Arthralgia	3 (10)	0
Eosinophilia	2 (6.7)	0
Creatinine elevation	1 (3.3)	0
