# Supplementary material for: Clinical Outcome of Nivolumab Plus Ipilimumab in Patients with Locally Advanced Non-Small-Cell Lung Cancer with Relapse after Concurrent Chemoradiotherapy followed by Durvalumab
Source: Cancers (Basel). 2024 Apr 3;16(7):1409. doi: 10.3390/cancers16071409 (PMC11011053; doi:10.3390/cancers16071409)
Supplement: Supplementary file 1 [file cancers-16-01409-s001.zip › Supplementary Table S3.pdf]

Supplementary Table S3. Comparison of adverse events with durvalumab and nivolumab plus ipilimumab

Number of patients	Durvalumab		Nivolumab plus ipilimumab	
	Adverse event	Grade	Adverse event	Grade
1	Arthralgia	2	Arthralgia	2
	Pneumonitis	2	-	
2	Pneumonitis	2	-	
3	-		Liver dysfunction	1
4	Pneumonitis	1	Skin disorder	2
5	Eosinophilia	1	Pneumonitis	3
	-		Liver dysfunction	2
6	Liver dysfunction	2	Liver dysfunction	1
	Pneumonitis	1	-	
7	Pneumonitis	2	Hypothyroidism	2
8	Pneumonitis	2	Hypothyroidism	1
	-		Skin disorder	3
	-		Diarrhea	2
9	Pneumonitis	3	Liver dysfunction	1
10	Skin disorder	1	Arthralgia	1
	-		γ-GTP increased	1
	-		ALP increased	1
11	-		Diarrhea	1
12	Pneumonitis	2	Pneumonitis	1
	-		Liver dysfunction	1
13	Pneumonitis	2	-	
14	Skin disorder	1	Skin disorder	2
	Pneumonitis	1	Hypothyroidism	1
15	Pneumonitis	1	Liver dysfunction	1
	Eosinophilia	1	-	
16	Arthralgia	2	Arthralgia	1
	Pneumonitis	2	Diarrhea	3
	-		Skin disorder	1
17	Pneumonitis	1	-	
18	Pneumonitis	3	-	

19	Hypothyroidism	2	Hypothyroidism	2
20	Hypothyroidism	1	-	
21	-		-	
22	-		Skin disorder	1
23	Hypothyroidism	2	Hypothyroidism	2
	Skin disorder	1	Arthralgia	2
24	Pneumonitis	3	-	
25	Arthralgia	1	-	
26	Hyperthyroidism	1	Skin disorder	2
27	Cr elevation	1	Stomatitis	1
	-		Shingles	2
	-		Eosinophilia	1
28	Pneumonitis	1	Pneumonitis	2
29	Pneumonitis	1	-	
	Skin disorder	1	-	
30	Pneumonitis	1	-	

ϒ-GTP, ϒ-Glutamyl transpeptidase; ALP, alkaline phosphatase. Cr, creatinine
